# The Story of the Insane from Year to Year

**Published:** 1894-10-06

**Authors:** 


### THE STORY OF THE INSANE FROM YEAR TO YEAR.

(7th Series.)

### THE TEZPUR ASYLUM, ASSAM.

From the report presented by Surgeon-Colonel Warburton we learn that this asylum contained on December 1st, 1893, 99 patients, of whom 71 were males and 29 females; but the latter number is probably a misprint for "28." There are 22 criminal lunatics; and the nationalities of the total of 99 are: Coolies, 27; other foreigners 20, and Assamese 52. The recoveries reached the fair percentage of 32·55 of the admissions, and the deaths were only 4·44 of the average number resident. Turning to the table showing the causes of the insanity, we find spirit drinking occupying a prominent place, and hereditary influence is also mentioned, while others are given which we rarely see in England, such, for instance, as opium eating and the use of various forms of Indian hemp. There were 15 deaths. The asylum is under the superintendence of Surgeon-Major Macnamara, and the Chief Commissioner observes that the condition and management of the asylum reflect the highest credit on him.

### HULL BOROUGH ASYLUM.

This asylum is something more than full, and it increased its numbers during 1893 by 14, there being 370 in residence at the close of the year. There were 115 admissions, 34 recoveries, and 35 deaths. The recoveries were 29·82 of the admissions, and the deaths were 7·43 of the average number resident. Intemperance caused the insanity in 37 of the admissions, and hereditary influence in 45; so that these fertile and preventible causes are responsible for 82 of 115 admissions. The ratepayers of Hull should think this over, and they will probably arrive at the conclusion that it is time something was done to stem this tide of insanity which we are allowing to flow in on us in all directions, and of which the ebb is far off indeed unless stringent measures are taken. It is proposed to enlarge the asylum so that more than 500 lunatics may be housed, and it will not stop there. From one end of England to the other we hear the same story—want of accommodation everywhere, but of prevention of the disease scarcely a word anywhere. Table V. shows that 27 of the deaths were due to cerebral and spinal diseases, and that only three were due to consumption. Dr. Merson says that 48 of the admissions were, from the nature of their disease, incurable, and of the remainder scarcely one-third presented any reasonable hope of recovery. The committee "express their satisfaction with the manner the patients have been looked after and their wants attended to by the medical superintendent."

### WORCESTER COUNTY ASYLUM.

Here there was an increase in the numbers during the year from 901 to 1,003. The admissions were 246, the recoveries 64, and the deaths 72. The recoveries were 41·5 per cent. on the admissions, and the deaths 7·5 on the average number resident. Hereditary influence or predisposition had something to do with the cause of the insanity in 63 of the admissions, and intemperance in drink is the assigned cause in 23. Cerebral and spinal diseases caused death in 26 instances, and tubercular diseases in 10. Like a good many other medical superintendents, Dr. Cooke has got into trouble over those miserable "continuation orders," and he details a curious case in which the order lapsed when the patient was absent on trial. The patient relapsed, and was sent back to the Asylum five days before the second anniversary of the date of his reception order, and as the "continuation order" must be sent to the Commissioners at least seven days before the original order expires, the patient had to be re-certified. There is no duty so irksome as a useless duty, and it would seem as if medical officers in asylums were to be burdened with this useless duty for a long time to come, although why they should be compelled to suffer for the stupidity of others it is not easy to see. Dr. Cooke has not lost sight of the advantages accruing from the training of attendants and nurses. We are pleased to note that during the past year the scope of instruction has been extended, and that lectures have been delivered by the assistant medical officers. Dr. Cooke hopes the Committee will offer prizes to those of the staff who do best at the examinations. We join in this hope, and we add another hope that the district auditor will see the thing in the same light as Dr. Cooke, otherwise he may construe "prize" to mean "gratuity," and it is questionable whether a gratuity can be legally given out of the rates. The Committee "have much satisfaction in being able to record that Dr. Cooke continues to discharge the very responsible duties of his position with the same care and attention to the interests of the patients and of the asylum which he has done since he first filled his office." The weekly cost of maintenance is 8s. 4d. per head.

### ROXBURGH DISTRICT ASYLUM.

The numbers increased from 236 to 247. There were 63 admissions, 25 recoveries, and 10 deaths. The recoveries stand at 39·6 of the admissions, and the deaths at 4·1 on the average number resident. The recoveries are almost exactly the average of kindred institutions, and the deaths are much below the average. Intemperance in drink is given as the cause of the insanity in 15 of the 63 admissions, and hereditary tendency in 14. "Previous attacks" figure in 26, and in only 5 is the cause "unknown." Two of the 10 deaths were caused by general paralysis and 3 by consumption. In many asylums the supply of water ran short during the last summer, but few medical superintendents were put to such straits as Dr. Johnstone. "It was found necessary," he says, "to discontinue the bathing of the patients, to cease cleansing the floors, to restrict the washing of clothes, to cut off the water n the lavatories during the greater part of the day, and to shut up many of the water-closets." A medical superintendent's life has always strong veins of worry and anxiety in it, and any occurrence like a deficiency of water adds terribly to these. We notice that five of the staff have obtained the nursing certificate of the Medico-Psycological Association. The rate of maintenance is £27 per head per annum.

### SUSSEX COUNTY ASYLUM, HAYWARD'S HEATH.

On January 1st, 1893, there were in this asylum 857 patients, and on December 31st the number had fallen to 828. The admissions were 298, the recoveries 68, and the deaths 84. Only 19 of the admissions are returned as due to intemperance, and 36 to hereditary influence, but as the cause is stated to be "unknown" in 131 instances the table is practically useless. The recoveries stand at 27 per cent. on the admissions and the deaths at 9·6 on the average number resident. Of the deaths, 40 were owing to brain diseases, nine of these being from progressive paralysis of the insane, and 12 of the other deaths were from phthisis. The Medical Superintendent's report covers the space between September 30th, 1892, and September 30th, 1893, while the statistical tables show the changes which occurred in the asylum from January 1st to December 31st. No good end is served by this mixture, and it is to be hoped another year will see the resulting confusion remedied. Post-mortem examinations were made in 27 cases, and Dr. Saunders regrets that no more were made, but he adds that the personal feelings of the living are not matters which can be subordinated to rules nd regulations. Here we are in full accord with Dr. Saunders, and we again express disapproval of the wholesale post-mortem making which some asylums boast of, and which must have caused much pain to the living. Owing to pressure on the accommodation, the sanatorium had to be occupied by female patients, and two days afterwards a case of scarlet fever appeared in the main building. Provision was found for this case in an empty cottage, but the occurrence shows the necessity for an infectious hospital. Gardener Stebbings, Attendant Parkes, and Laundress E. Pinyoun received pensions during the year, and we congratulate all these officials on having obtained reward for long service and provision for old age. The committee say that Dr. Saunders and the other officers have performed their duties in a highly satisfactory manner. The weekly rate of maintenance is 9s. 6d. per head.

